# Multilocus microsatellite markers for molecular typing of *Candida tropicalis* isolates

**DOI:** 10.1186/s12866-014-0245-z

**Published:** 2014-11-20

**Authors:** Yuan Wu, Hai-jian Zhou, Jie Che, Wen-ge Li, Fu-ning Bian, Shuan-bao Yu, Li-juan Zhang, Jinxing Lu

**Affiliations:** State Key Laboratory for Infectious Disease Prevention and Control, Collaborative Innovation Center for Diagnosis and Treatment of Infectious Diseases, National Institute for Communicable Disease Control and Prevention, Chinese Center for Disease Control and Prevention, Chang bai Road 155, Chang ping District Beijing, China; Department of Gynecology and Obstetrics, Beijing Obstetrics and Gynecology Hospital, Capital Medical University, Beijing, China

**Keywords:** *Candida tropicalis*, Microsatellite markers, Population structure, Molecular typing

## Abstract

**Background:**

*Candida tropicalis* is considered to be the leading pathogen causing nosocomial fungemia and hepatosplenic fungal infections in patients with cancer, particularly those with leukemia. Microsatellite-based typing methods using sets of genetic markers have been developed and reported for population structure analysis of *C. albicans*, *C. glabrata*, and *C. parapsilosis*, but no studies have been published for genetic analysis of *C. tropicalis*. The objective of this study was to develop new microsatellite loci that have the ability to distinguish among *C. tropicalis* isolates.

**Results:**

DNA sequences containing over 10 bi- or tri-nucleotide repeats were selected from the *C. tropicalis* genome database. Thirty PCR primers sets specific for the microsatellite loci were designed and tested using eight clinically independent isolates. According to the amplification efficiency, specificity, and observed polymorphisms, eight markers were selected for further population structure analysis and molecular typing. Sixty-five independent *C. tropicalis* isolates were genotyped using these 8 markers. Based on these analyses, six microsatellite loci were confirmed, although two loci were found to be with unstable flanking areas. The six polymorphic loci displayed 4–22 alleles and 7–27 genotypes. The discriminatory power of the six loci ranged from 0.70 to 0.95. Genotyping results obtained by microsatellite analysis were compared to PCR-fingerprinting and multi-locus sequence typing (MLST). The comparisons showed that microsatellite analysis and MLST had the similar discriminatory power for *C. tropicalis*, which were more powerful than PCR-fingerprinting.

**Conclusions:**

This is the first attempt to develop new microsatellite loci for *C. tropicalis*. These newly developed markers will be a valuable resource for the differentiation of *C. tropicalis* isolates. More *C. tropicalis* isolates will need to be sequenced and analyzed in order to fully show the potential of these newly developed microsatellite markers.

## Background

With the increasing number of immunocompromised patients, long-term hospitalized patients, and invasive medical conditions and therapy, the genus *Candida* has emerged as a major group of opportunistic pathogens that cause both superficial and invasive infections in humans [1,2]. *Candida* is considered to be the fourth most commonly isolated organism from nosocomial bloodstream infections in United States and the sixth most common in Europe [3-5]. Invasive infections caused by *Candida* species are associated with significant morbidity and mortality [6]. Although *C. albicans* accounts for the majority of infections, other non- *albicans Candida* species such as *C. tropicalis* have increasingly been recognized as important human pathogens. *C. tropicalis* is the leading pathogen causing nosocomial fungemia and hepatosplenic fungal infections in patients with cancer, particularly leukemia patients [7]. *C. tropicalis* is the second most frequently isolated non-albicans pathogen in the Asia-Pacific region and in Brazil [8]. In large independent epidemiologic surveys, the isolation rate of *C. tropicalis* from blood was shown to be 5-30% [9]. In evolutionary terms, this species is closely related to *C. albicans* [10]. Previous studies conducted in Asia show an intermediate frequency of fluconazole resistance for *C. tropicalis* strains, which was originally observed in *C. glabrata* isolates [11,12]. Furthermore, a high proportion of *C. tropicalis* isolates exhibits low susceptibility to flucytosine [13,14].

Although several molecular typing methods have been used to determine the molecular epidemiology and resistance of *C. tropicalis*, such as MLST [15], randomly amplified polymorphic DNA (RAPD) [16,17] and pulsed field gel electrophoresis (PFGE) [18,19], population structures and genetic investigations for *C. tropicalis* have not been as extensive as they have been for *C. albicans* studies. MLST reveals different geographical origins, anatomic sources, and other characteristics between clades of closely related isolates [15]. Furthermore, some isolates of *C. tropicalis* have been shown to be associated with anti-fungal resistance [11]. Fifty-two diploid sequence types (DSTs) from China were recently generated and added to the global MLST database [20]. RAPD is considered to be a promising tool for yeast genotyping, especially when used with different primer combinations [21]. However it has some limitations for population structure analysis because it relies on a large intact DNA template sequence that hinders reproducibility [17]. Microsatellites are defined as short tandem repeats of two to six nucleotides, known to be highly polymorphic and have been widely used for polymorphism analysis of fungi [22,23]. Microsatellite provides an alternative typing scheme because it is an easy-to-perform and reproducible method suitable for large-scale studies of *C. tropicalis* epidemiology. Microsatellite-based typing methods using sets of genetic markers have been developed and reported for population structure analysis of *C. albicans* [2,24], *C. glabrata* [25,26], and *C. parapsilosis* [27,28], but no studies have been published for genetic analysis of *C. tropicalis*.

The aim of this study was to develop a microsatellite-based typing method using a new set of six markers for population genetic analysis of *C. tropicalis*. The polymorphism of microsatellites was evaluated by PCR and allele sizing of 65 *C. tropicalis* isolates. Our results indicate that the discriminatory power (DP) of the 6 loci ranges from 0.70-0.95, illustrating this to be a useful method for genetic studies of *C. tropicalis*.

## Results

### Screening and selection of repeat regions in *C. tropicalis* genome sequence database

We searched the *C. tropicalis* genome using Tandem Repeat Finder (TRF) software and generated over 4,000 sequences whose repeated motif was at least 1 bp. The criteria defined were that the sequence should contain at least 10 repeats with equal or greater than 2 bp in the core motif. In total, 30 microsatellite loci were selected. These sequences have a high probability of showing greater genetic variability, are likely located outside known coding regions, and dispersed evenly throughout the genome. To evaluate the effectiveness of the 30 loci, 30 pairs of specific primers were designed and genomic DNA from 8 *C. tropicalis* isolates was used as a template for PCR. After removing the unsuccessful and non-polymorphic loci, eight microsatellite markers were chosen for further microsatellite analysis (Table 1). Locus-specific primers were then designed for these markers. The forward primers were fluorescently labeled with 6-carboxyfluorescein (FAM), 6-carboxyhexafluorescein (HEX), 5-carboxy-x-rhodamine (ROX), or 6-carboxytetramethylrhodamine (TRMRA) (Table 1).

**Table 1:** **Microsatellite DNA sequences selected, sequences, and primers**

**Microsatellite designation**	**Primer sequence**	**Repetitive motif**	**Range of PCR fragment size (bp)**	Fluorescent label*
Ctrm1	F: CAACAGTTGATAGATCAAGC	(AGA) _22_	370-454	FAM
	R: CGAACTATCACTTTTAGGAG			
∆Ctrm7	F: GACTCTGAATCGGTTTTGTG	(CA)_13_	286-322	HEX
	R: CGCTCATTCTCATAATCACT			
Ctrm10	F: AGTTTTCCTGTTGCTGGTTG	(ATG)_52_	315-370	ROX
	R: CATTGAGATTGGAAGAAGTG			
Ctrm12	F: TGTGTGTCTATTACCTCCCA	(AC)_39_	234-263	FAM
	R: CTGTCAGTTGTACATCATCG			
∆Ctrm15N	F: CCCTACTAGGACCTCCACCG	(CAA)_16_	365-392	TAMRA
	R: AAAGAATGGCGATGAAGTTG			
Ctrm21	F: TGTGTCTTGTAAAAGCCACC	(TG)_22_	328-363	HEX
	R: GGATTACTGGACTTGACCTG			
Ctrm24	F: ACAACTACTGACATCCCAGC	(TA)_13_	439-454	ROX
	R: CTTCAGTATTCACCCCTTTC			
Ctrm28	F: TAGTTCGAATTTGTTTGGAT	(TTA)_12_	397-406	TAMRA
	R: GTAAAGTCACGGGGTATTGT			

∆ In the expanded microsatellite analysis of 65 *C. tropicalis* isolates, these 2 loci showed unstable flanking sequences and were excluded for further population structure analysis.

*FAM: 6-carboxyfluorescein, HEX: 6-carboxyhexafluorescein, ROX: 5-carboxy-x-rhodamine, or TRMRA: 6-carboxytetramethylrhodamine.

### Microsatellite analysis

**Figure 1:**
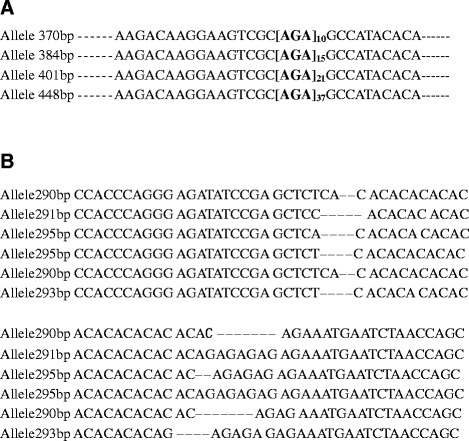
**Alignment of parts of the different alleles’ sequences. A**. Parts of the sequences of the different alleles of the Ctrm1 marker showing the numbers of microsatellite repeats. For allele numbers and frequencies, refer to Table 2. **B**. Parts of the sequences of the different alleles of the Ctrm7 marker showing the numbers of microsatellite repeats with unstable flanking area.

In order to evaluate the specific amplifications and polymorphisms, the microsatellites selected were used to type 65 clinically independent *C. tropicalis* isolates. *C. tropicalis* is a diploid species, therefore one or two PCR fragments per locus were obtained for each strain, and each fragment was assigned as a unique allele. Isolates presenting two PCR products were typed as heterozygous, while strains having a single PCR fragment were typed as homozygous. One allele of each length was sequenced. For most of the microsatellite markers, a direct correlation between the fragment size and the number of microsatellite repeats was found, with the differences in fragment sizes being consistent with the variation in the number of repetitions (Figure 1A). However, for few alleles, we sequenced repeatedly to obtain the correct number of repeats. In the case of Ctrmm7 and Ctrmm15N loci, several isolates of these alleles were sequenced and analyzed using BLAST, which showed that they contain unstable flanking areas, such as a deletion (Figure 1B). Six loci (Ctrmm1, 10, 12, 21, 24, and 28) were used for final population structure and genetic analysis. The characterizations of the loci selected are illustrated in Table 2. In total, 22, 12, 12, 5, 4 and 15 alleles were found for the Ctrm1, 10, 12, 21, 24 and 28 loci, respectively (Table 2). The analysis of the 65 isolates revealed all microsatellite loci to be polymorphic, showing 4 to 22 alleles from 7 to 27 distinct genotypes (Table 2). The detailed information of alleles and the corresponding number of repeats are shown in Table 3. The differences in length for the 6 markers were due to a varying fold repeat of a hexanucleotide (Figure 1A). The DP for each marker was calculated according to the Simpson index (Table 2) [29] as follows: $DP=1-\frac{1}{N\left(N-1\right)}{\displaystyle \sum_{j=1}^Snj\left(nj-1\right)}$ where *N* is the number of strains, *s* is the total number of different genotypes, and *nj* is the number of strains of genotype *j* [29]. The results show that Ctrmm1, producing 22 different alleles and 27 genotypes, was the microsatellite with the highest DP (0.95), while Ctrmm28 presented the lowest DP (0.70), with different 15 alleles and 7 genotypes (Table 2). The total number of different alleles and genotypes and the respective frequencies obtained for all microsatellite markers examined in Table 2. Amplification products were observed for all 65 *C. tropicalis* strains at all 6 loci, showing powerful typing ability in all cases except for loci Ctrmm21, Ctrmm24, and Ctrmm28, with 3, 4, and 4 strains of unsuccessful PCR amplification respectively (Table 3). The amplifications were performed in triplicate. The particular strain numbers and repeated motif numbers of each allele are summarized in Table 3. The sequencing of alleles allowed us to determine that the exact length of the PCR products. For example, alleles 328 and 332 for Ctrmm21 are composed of 8 (329 bp) and 9 (331 bp) repeats (Table 3).

**Table 2:** **Characteristics of microsatellite loci selected**

**STR**	**No. of alleles**	**No. of genotypes**	DP*	**Allele frequency**	**Genotype frequency**	**%Heterozygosity**
Ctrm1	22	27	0.95	0.008-0.154	0.02-0.14	75.4
Ctrm10	12	16	0.91	0.008-0.285	0.02-0.23	70.8
Ctrm12	12	13	0.85	0.008-0.354	0.02-0.34	73.8
Ctrm21	5	16	0.91	0.016-0.290	0.02-0.19	73.8
Ctrm24	4	7	0.78	0.016-0.631	0.02-0.39	44.6
Ctrm28	15	7	0.70	0.082-0.566	0.02-0.54	16.9

*DP, discriminatory power.

**Table 3:** **Number of repeats of the six markers for the 65** ***C*** **.** ***tropicalis*** **isolates**

**Marker**	**Allele size (bp)**	**No. of isolates**	**No. of repeats**
Ctrm1	370	8	10
	373	2	11
	376	1	12
	382	2	14
	384	2	15
	387	14	16
	390	11	17
	393	12	18
	396	2	19
	398	14	20
	401	8	21
	404	5	22
	407	20	23
	410	6	24
	413	6	25
	416	2	26
	419	1	27
	422	1	28
	440	1	34
	448	3	37
	451	4	38
	454	5	39
Ctrm10	315	19	7
	318	3	8
	322	37	9
	325	5	10
	331	29	12
	334	19	13
	340	2	15
	344	1	16
	353	3	19
	356	7	20
	361	4	22
	370	1	25
Ctrm12	234	4	12
	236	44	13
	238	46	14
	242	4	16
	244	10	17
	248	5	19
	250	8	20
	252	2	21
	254	4	22
	257	1	25
	259	1	26
	263	1	28
*Ctrm21	0	3	0
	328	33	8
	329	7	8
	331	36	9
	332	25	9
	334	2	13
	353	3	20
	355	9	21
	357	4	22
	363	5	25
*Ctrm24	0	4	0
	439	2	10
	444	20	12
	448	14	14
	450	77	15
	454	9	17
*Ctrm28	0	4	0
	397	30	9
	400	10	10
	403	69	11
	406	13	12

*In these 3 markers, there were 3 or 4 unsuccessful amplifications.

### Population structure study of *C. tropicalis* using multiple typing methods

**Figure 2:**
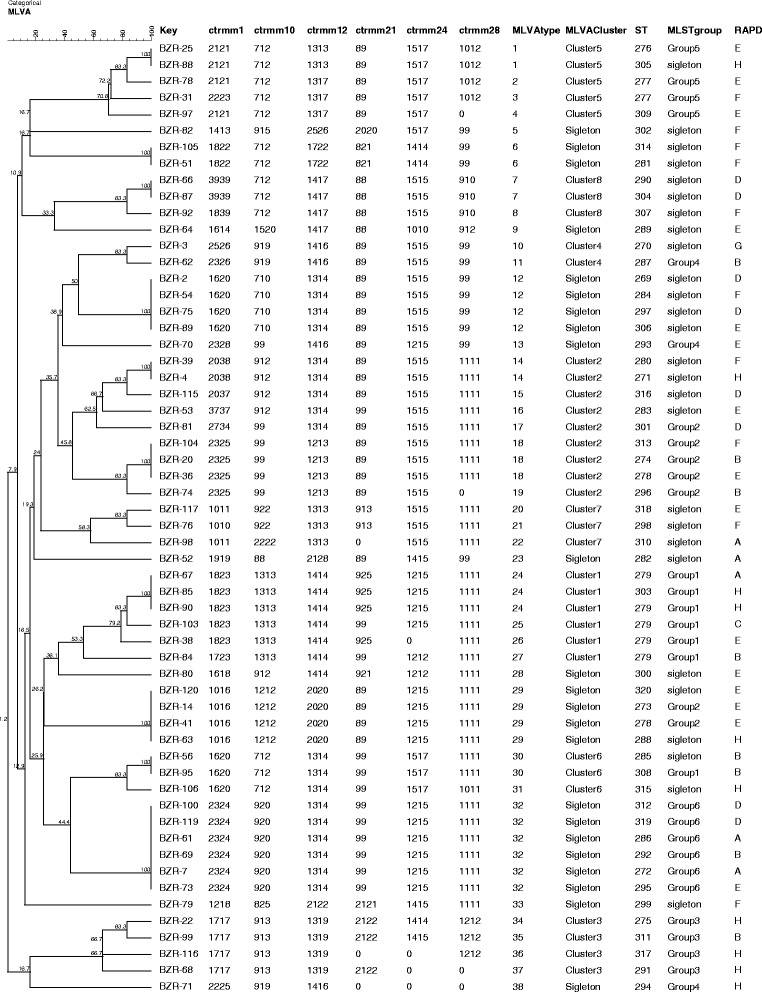
**Cluster analysis of 58** ***C*** **.** ***tropicalis*** **based on 6 microsatellite loci by the use of Bionumerics version 4.0 software and comparisons among STR, MLST and RAPD.** The numbers below each microsatellite number are their allelic profiles. For example, 2121 of ctrmm1 means 21 repeats of the allele and indicates this stain is homozygous; 2223 of ctrmm1 means 22 repeats of one PCR fragments, and 23 repeats of the other allele, which indicating the stain is heterozygous. As for 712 of ctrmm10, it means 7 repeats and 12 repeats for the two allele of the strain, while number 99 of ctrmm10 means 9 and 9 repeats for the two allele of the strain. Number 0 show the unsuccessful amplification of those markers for few strains.

**Figure 3:**
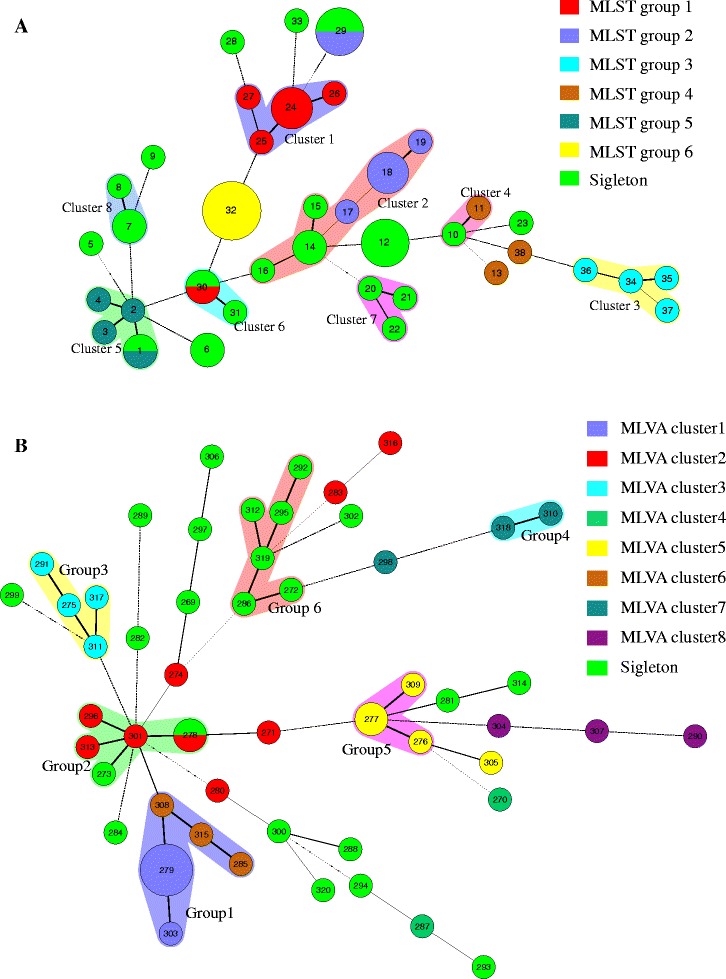
**Minimum spanning tree analysis based on MLVA and MLST. A**. Minimum spanning tree analysis of 58 *C*. *tropicalis* based on allelic profiles at 6 microsatellite loci. **B**. Minimum spanning tree analysis of 58 *C. tropicalis* based on allelic profiles of MLST data. The group differences between STR and MLST were shown directly in the figure. Each circle corresponds to a repeat type, the number of which is indicated inside the circle. The colors of the halo surrounding the repeat types denote type that belong to the same cluster. The lines between circles indicate the similarity between profiles (bold, 5 alleles in common; normal, 4 alleles; dotted, ≤3 alleles).

Fifty-eight of the *C. tropicalis* isolates typed by MLST and PCR fingerprinting analysis were selected for genotyping using the microsatellite markers so that the results of these three typing methods could be compared. Based on the microsatellite loci database, an unweighted pair group method (UPGMA) tree was constructed based on genetic distances (Figure 2), in which the genotyping type for each strain by the other two methods was also shown. Eight distinct short tandem repeat (STR) clusters were constructed (Figures 2 and 3), while 6 MLST groups and 4 RAPD groups were produced (Figure 2). Microsatellite analysis detected 86 different genotypes, whereas MLST detected 70 genotypes and RAPD detected 20 genotypes. Therefore, it could be deduced that STR analysis and MLST were both found to have a high capacity to discriminate isolates, and a relatively high level of concordance between the results was displayed (Figure 3). For better understanding of the DP of MLST and microsatellite for *C. tropicalis*, we constructed minimum spanning trees (MSTs) based on microsatellite allele profile (Figure 3A) and MLST allele profile (Figure 3B). MLVA cluster 1 is composed of strains predominant in MLST group 1. MLVA cluster 2 contains strains from MLST group 2 and some singletons (Figure 3A). MLVA cluster 4 has only 2 strains, one from MLST group 4, and the other is singleton (Figure 3A). MLVA cluster 6 includes 2 singletons and 1 from MLST group 1 (Figure 3A). For cluster 7 and 8, they are totally composed of singletons (Figure 3A). While strains in MLST groups 3 and 5 were also in STR cluster 3 and 5 (Figure 3A). One strain in MLST group1 is separated far away and included in cluster 6 (Figure 3A). The same situation happened to MLST group 2, in which one strain is split as singleton (Figure 3A). For MLST group 4, all its strains are separated as independent ones (Figure 3A). In figure 3B, trees are built on MLST allele profile and its corresponding relationship with MLVA cluster is shown. Strains from MLVA cluster 1 and cluster 6 clustered as MLST group 1 (Figure 3B). For group 2, 2 singletons and strains in MLVA cluster 2 are included (Figure 3B). Group 3 are totally composed of the same strains in cluster 3 (Figure 3B). Group 4 is made up with 2 strains in MLVA cluster 7 (Figure 3B). Strains in group 5 are all in MLVA cluster 5 (Figure 3B). Group 6 is comprised completely by singletons (Figure 3B). Strains in cluster 2 disperse widely in MLST MST (Figure 3B). Strains from cluster7 and cluster 5 are separated as singleton independently (Figure 3B). Isolates BZR-71 were separated far away from BZR-62 and BZR-70 of MLST group 4 (Figure 2). No relationships were found between genotypes and specimen type, hospital origin, and fluconazole resistance in either typing method. The differences in size polymorphisms of microsatellite analysis indicate that microsatellites appear to be evolving with a higher rate of sequence divergence and may be helpful for driving deeper establishment of unrelated profiles, which could be useful in outbreak situations but less effective for the determination of long-term genetic relatedness [23].

### Reproducibility and statistics

For all the strains tested, the microsatellite types were the same for analysis the same or different DNA from same strains in different runs.

## Discussion

*C. tropicalis* is a diploid organism similar to *C. albicans*. A variety of strain typing methods have been used for the differentiation of the *C. tropicalis* family, including PCR fingerprinting [21], MLST [11], and PFGE [18]. Shu-Ying Li *et al.* [11] compared MLST *via* PFGE for population structure and genetic relationship analysis of clinical *C. tropicalis* isolates. They found that the genetic profiles of *C. tropicalis* clinical isolates obtained by these two methods were highly correlated, in which MLST was slightly less discriminatory than PFGE. In addition, fluconazole-resistant *C. tropicalis* isolates were grouped into a clonal cluster in both MLST and PFGE [11]. Another report showed that most of the tested *C. tropicalis* isolates were assigned to a single large recently evolved group that contained several small clonal clusters. It indicates that *C. tropicalis* resembles *C. albicans* phylogenetically. Such evolution pattern could be explained as a predominantly clonal mode of reproduction but with a frequency of recombination events high enough to generate a population with characteristics similar to a sexually reproducing species [30]. In a previous study, we discovered new MLST types of *C. tropicalis* from mainland China, showing several independent groups when compared to the global *C. tropicalis* MLST database [20]. It had been considered that RAPD was a promising tool for yeast genotyping, especially when used with multiple primer combinations, by which subtypes are found to be related to their geographic origin, evolutionary and taxonomic classification [21]. However, with the development of newer molecular methods, RAPD is now considered as unstable and is not easy to standardize. Microsatellites are found in all genomes and are increasingly being used as molecular marker [23]. The microsatellite method is discriminatory, reproducible, and easy to perform. Furthermore, the results remain stable over many generations [23]. Microsatellite genotyping has been successfully used to characterize and rapidly type isolates of several yeast species including, *Aspergillus fumigatus* [31], *Saccharomyces cerevisiae* (14), *Penicillium marneffei* [32], *C. albicans* [33], *C. krusei* [34], *C. parapsilosis* [27], *C. glabrata* [35] and *Cryptococcus neoformans* and *gattii* [36,37].

To our knowledge, this is the first report developing and testing microsatellite markers for *C. tropicalis*. We screened the genome of *C. tropicalis* for microsatellites. After testing of the candidate loci, six markers were selected. All six loci display highly polymorphism rates and discriminatory power. Ctrmm1, 10 and 12 markers are the most discriminatory microsatellites, with 100% amplification efficiency, high discriminatory power and varying repeats of the same motif (Table 2). Sequencing confirmed that the length of polymorphisms were due to the number of nucleotide motif repeats. For the other markers Ctrmm21, 24 and 28, irregular correlation between repeat motif and sequence size for some alleles were observed. Therefore, we sequenced these uncertain alleles to obtain the accurate length. The DP of these three, Ctrm21, 24 and 28, vary between 0.70-0.91. For marker Ctrm21, 24 and 28, there were 3–4 isolates of unsuccessful PCR amplification. We repeated three times for these amplifications. Nucleotide mutation in the flanking area of those markers may lead to unspecific combination of primers to template DNA. The second reason may be that the template DNA area for designing primers is not so conserved for isolates with wide origins. With more sequences of *C. tropicalis* released, a more conserved area may be developed. In summary, the primers coverage ability and the DNA structure changes may contribute to the unsuccessful amplification. Ctrm1 loci displayed the highest DP and heterozygosity, while the Ctrm 28 showed the lowest heterozygosity and DP. Ctrmm1, 10 and 12 markers displayed 100% amplification efficiency in this study using strains with limited geographic distribution. Compared with polymorphic microsatellite loci (*EF3*, *CDC3*, *HIS3*, *ERK1*, *2NF1*, *CCN2*, *CPH2*, *EFG1*, *CAI* AND *CAIII* to *CAVII*) used for *C. albicans* [38,39], whether they are efficient to distinguish world-wide strains still need further analysis. And with finish of more *C. tropicalis* whole genome, more microsatellite markers will be selected.

In our study, MLST and PCR-fingerprinting methods were used to evaluate the six microsatellite markers newly developed for studying the population structure, genetic relativity, and molecular epidemiology of *C. tropicalis* isolates from various geographic and anatomic sites. Our data indicates that MLST and microsatellite analysis appear to have similar potentials to differentiate *C. tropicalis* and both have discriminatory power superior to RAPD analysis. It is well known that RAPD is a conventional DNA-based typing method, while microsatellite and MLST are exact DNA-based typing methods [39]. The interpretation of RAPD patterns is based on the number of size of the amplified fragments, and banding patterns are easily effected by kinds of experimental conditions [39]. The drawbacks of RAPD are its reproducibility and data comparison between labs. Both microsatellite and MLST generate unambiguous results with an excellent reproducibility, which could be exchanged and compared globally [39]. Although there was high agreement between the methods for the assignment of genotypes, disagreement of clustering of unrelated isolates was also observed (Figure 3). Some singletons in the MLST analysis formed new groups using the STR method. Furthermore, strains clustered in MLST groups were separated and formed new STR clusters with other isolates, such as MLST group 1, 2 and 4 (Figure 3).

In conclusion, these six new microsatellites are a valuable tool for the differentiation of *C. tropicalis* isolates and will have a strong application in studies that must distinguish epidemiologically related isolates, such as nosocomial cross-transmission analyses, and the study of kinetics of the colonization-to-infection process. The standardization of the microsatellite typing systems and the creation of public databases that would make microsatellite allele data available worldwide are essential issues that deserve attention and resources. The overall higher similarity level of gene sequences from *C. tropicalis* as compared with *C. albicans* may indicate that many more isolates are needed to be sequenced to reveal full strain diversity in *C. tropicalis*, or to discover a strain type well adapted to humans.

## Conclusions

*C. tropicalis* is considered to be the leading pathogen causing nosocomial fungemia and hepatosplenic fungal infections in patients with cancer, particularly those with leukemia. Several molecular typing methods have been used for studying *C. tropicalis*, but no study of microsatellite analysis has been published for genetic analysis of *C. tropicalis*. In this study, we firstly developed new microsatellite loci for *C. tropicalis*. The six loci selected showed high discriminatory power, similar discriminatory ability with MLST and more powerful than PCR-fingerprinting. These newly developed markers will be a valuable resource for the differentiation of *C. tropicalis*. More *C. tropicalis* isolates will need to be sequenced and analyzed in order to fully show the potential of these newly developed microsatellite markers.

## Methods

### Isolates and DNA extraction

To evaluate the DP of the microsatellite markers, 65 clinical *C. tropicalis* isolates from different anatomical sites were genotyped. All isolates were collected from adult patients over a 1-year period of several hospitals in China, covering both male and female patients of varying ages. Most of these isolates have been done MLST analysis, showing diverge DST types. The samples were from clinical routine inspection and patients were informed and provided consent. Sample collection is coincided with the protocol of the hospital and is approved by China-Japan Friendship Hospital Ethics Committee. ATCC 750 was analyzed as reference strain. The specificity of the primers was checked by studying the following references strains: *C. albicans* ATCC 753, *C. glabrata* ATCC 2001, *C. parasilosis* ATCC 10232, *C. kefyr* ATCC 4135, *Saccharomyces cerevisiae* ATCC 10668, *C. krusei* CGMCC 2.1848 (China General Microbiological Culture Collection Center, Beijing). All isolates were identified by internal transcript sequence (ITS) sequencing and AUX 20C (BioMe´rieux, France). The universal primers ITS1 and ITS4 [40] were used to amplify the ITS fragment and to sequence it bi-directionally. The strains were stored at −80°C in brain–heart infusion media (Oxoid, UK). The isolates were maintained on Sabouraud glucose agar (SDA) (Oxoid, UK) during the study. Prior to DNA isolation, yeast cells were grown on SDA for 24 h at 37°C. Genomic DNA of the isolates was extracted using a Yeast DNA Purification Kit (Tiangen, China), according to the manufacturer’s protocol. DNA concentrations were estimated with a spectrophotometer absorbance at 260 nm. DNA extracts were stored at −20°C.

### Microsatellite selection, PCR primer design and amplification

A search of *C. tropicalis* genome sequences available in GenBank (Accession number: AAFN00000000.2) was performed to identify repeat sequences using the TRF software from Gary Benson (http://tandem.bu.edu). Thirty microsatellites containing over 10 bi-or tri-microsatellite repeat units were selected, which were expected to have very high degrees of polymorphism. From these 30 pairs of primers specific for the non-variable flanking regions were designed for locus-specific amplification. Primer 5 software (http://www.premierbiosoft.com/primerdesign/) was used for the design of these primers. Genomic DNA of 8 *C. tropicalis* strains were used as template for typical PCR amplification. Amplification was carried out in a 50-μl volume containing 1 μl of *C. tropicalis* DNA. The composition of the PCR mixture was as follows: 5-μl 10 × PCR buffer, 0.25-μl rTaq polymerase (5U/μl, Takara.), 4-μl deoxynucleoside triphosphates mix (0.25 μM of each), 1-μl each primer (10pM), 10-μl 30% DMSO, and 27.75 μl ddH_2_O. After a 94°C preincubation step for 4 min, PCR amplifications were performed in total of 35 cycles under the following conditions: denaturation at 94°C for 45 s, annealing at 55°C for 45 s, and extension at 72°C for 40s, with a final extension step of 5 min at 72°C. PCR products were analyzed *via* 1.5% agarose gel electrophoresis. All PCR products were sequenced on both directions in order to confirm whether they were amplified correctly, and showed specific amplified polymorphism. The microsatellite markers with unsuccessful amplification and non-polymorphism were rejected. Eight microsatellite markers were selected for further analysis, which were distributed evenly throughout genome. The details of these 8 microsatellite markers are summarized in Table 1.

### Microsatellite and DNA sequence analysis

For the eight chosen microsatellite loci, PCR was performed with 65 clinical isolates to evaluate discriminatory power. The primers for these 8 selected loci were fluorescently labelled (Table 1), for further determination of alleles’ length by migration of the PCR products in a high resolution gel electrophoresis achieved by an automatic sequencer [38,39]. The PCR reaction volume was 20-μl, containing 2-μl 10 × PCR buffer, 1.6-μl deoxynucleoside triphosphates mix (0.25 μM of each), 0.1-μl rTaq polymerase (0.5U, Takara.), 0.4-μl each primer (10pM), 0.4-μl genomic DNA (40 ng), 4-μl 30% DMSO, and 11.1-μl ddH_2_O. After a 94°C preincubation step for 4 min, PCR amplifications were performed in the first 10 cycles under the following conditions: denaturation at 94°C for 45 s, annealing at 50-59°C for 45 s, reduction 1° in every cycle, and extension at 72°C for 1 min; and the second 25 cycles were as follows: denaturation at 94°C for 45 s, annealing at 50°C for 45 s, and extension at 72°C for 1 min, with a final extension step of 10 min at 72°C. The PCR of the loci was performed in two independent reactions: one was for Ctrm1, 7, 10, and 15 N; the other was for 12, 21, 24, and 28.

Products were analyzed *via* 1.5% agarose electrophoresis. Four groups of samples were then mixed for further analysis. We extracted 0.5-μl of the mixed amplification products and blended with 9-μl HIDI and 0.1-μl GS500LIZ. These mixtures were denatured at 95 °C for 5 min and rapidly chilled on ice. The samples were run using an ABI 3730XL genetic analyzer (Applied Biosystems). The sizes of the PCR products were determined using GeneMapper 4.0 software (Applied Biosystems). The alleles were then designed by their sizes (in base pairs). One allele of each length was sequenced in order to get the number of microsatellite sequence repeats. SeqMan software was used for sequence alignment (https://www.dnastar.com/).

### Population structure, PCR fingerprinting and MLST analysis

The allelic profiles of these 58 *C. tropicalis* strains were summarized and a dendrogram was then generated by UPGMA of the BioNumerics software version 5.1 (Applied Maths, Kortrijk, Belgium). The allelic profiles have been deposited in the Dryad database (http://doi.org/10.5061/dryad.8497b), and the DOI of data identifier is 10.5061/dryad.8497b. In order to compare the DP of MLST and microsatellite, and describe the relationships among isolates at the microevolutionary level, we performed allelic profile-based comparisons using a MST analysis with BioNumerics software. The MLST typing of those 58 *C. tropicalis* strains has been published by our research group previously. MST analysis links profiles so that the sum of the distances (number of distinct alleles between two sequence types, STs) is minimized [41]. Strains sharing the same allelic profile fall into the same circle, whose size is proportional to the number of strains with the profile. Clonal complexes were defined as groups of strains including a founder genotype and its corresponding single-locus variants. Clonal complexes are shown in shaded area in MST. The PCR fingerprinting and MLST method were performed as described previously [20,21].

### Reproducibility

The reproducibility of the microsatellite method was determined by analysis of the microsatellite genotypes by using the same or different DNA preparations obtained from the same isolated and assessed systematically by including a references strain as a control in each run.

### Statistical analysis

Allelic and genotypic frequencies were determined using ARLEQUIN (version 2.000) software and the DP of the markers was calculated as described by Hunter and Gaston [29].

## Abbreviations

MLST: Multi locus sequence typing
RAPD: Randomly amplified polymorphic DNA
PFGE: Pulsed field gel electrophoresis
DSTs: Diploid sequence types
TRF: Tandem repeat finder
FAM: 6-carboxyfluorescein
HEX: 6-carboxyhexafluorescein
ROX: 5-carboxy-x-rhodamine
TRMRA: 6-carboxytetramethylrhodamine
DP: Discriminatory power
